# Butyrylated starch intake can prevent red meat-induced O^6^-methyl-2-deoxyguanosine adducts in human rectal tissue: a randomised clinical trial

**DOI:** 10.1017/S0007114515001750

**Published:** 2015-06-17

**Authors:** Richard K. Le Leu, Jean M. Winter, Claus T. Christophersen, Graeme P. Young, Karen J. Humphreys, Ying Hu, Silvia W. Gratz, Rosalind B. Miller, David L. Topping, Anthony R. Bird, Michael A. Conlon

**Affiliations:** 1 Commonwealth Scientific and Industrial Research Organisation (CSIRO), Food and Nutrition Flagship, PO Box 10041, Adelaide BC, SA5000, Australia; 2 Flinders Centre for Innovation in Cancer, Flinders University of South Australia, Bedford Park, SA5042, Australia; 3 Rowett Institute of Nutrition and Health, University of Aberdeen, AberdeenAB21 9SB, UK; 4 Commonwealth Scientific and Industrial Research Organisation Mathematics, Informatics and Statistics, Glen Osmond, SA5064, Australia

**Keywords:** SCFA, Butyrate, DNA adducts, Resistant starch, Red meat, Fermentation, Microbiota

## Abstract

Epidemiological studies have identified increased colorectal cancer (CRC) risk with high red meat (HRM) intakes, whereas dietary fibre intake appears to be protective. In the present study, we examined whether a HRM diet increased rectal O^6^-methyl-2-deoxyguanosine (O^6^MeG) adduct levels in healthy human subjects, and whether butyrylated high-amylose maize starch (HAMSB) was protective. A group of twenty-three individuals consumed 300 g/d of cooked red meat without (HRM diet) or with 40 g/d of HAMSB (HRM+HAMSB diet) over 4-week periods separated by a 4-week washout in a randomised cross-over design. Stool and rectal biopsy samples were collected for biochemical, microbial and immunohistochemical analyses at baseline and at the end of each 4-week intervention period. The HRM diet increased rectal O^6^MeG adducts relative to its baseline by 21 % (*P*< 0·01), whereas the addition of HAMSB to the HRM diet prevented this increase. Epithelial proliferation increased with both the HRM (*P*< 0·001) and HRM+HAMSB (*P*< 0·05) diets when compared with their respective baseline levels, but was lower following the HRM+HAMSB diet compared with the HRM diet (*P*< 0·05). Relative to its baseline, the HRM+HAMSB diet increased the excretion of SCFA by over 20 % (*P*< 0·05) and increased the absolute abundances of the *Clostridium coccoides* group (*P*< 0·05), the *Clostridium* *leptum* group (*P*< 0·05), *Lactobacillus* spp. (*P*< 0·01), *Parabacteroides distasonis* (*P*< 0·001) and *Ruminococcus bromii* (*P*< 0·05), but lowered *Ruminococcus torques* (*P*< 0·05) and the proportions of *Ruminococcus gnavus*, *Ruminococcus torques* and *Escherichia coli* (*P*< 0·01). HRM consumption could increase the risk of CRC through increased formation of colorectal epithelial O^6^MeG adducts. HAMSB consumption prevented red meat-induced adduct formation, which may be associated with increased stool SCFA levels and/or changes in the microbiota composition.

Colorectal cancer (CRC) is one of the most frequently diagnosed malignancies worldwide, accounting for 10 % of all cancers and for approximately 20 % of all cancer-related deaths in developed countries^(^ ^1^ ^)^. Although there is a genetic component in CRC development, diet and other lifestyle factors are estimated to explain as much as 30–50 % of the global incidence of the disease^(^ ^2^ ^)^. According to the recent report from the World Cancer Research Fund and American Institute for Cancer Research (WCRF/AICR)^(^ ^2^ ^,^ ^3^ ^)^, there is convincing evidence that intake of red and processed meat increases the risk of CRC, whereas intake of dietary fibre is protective^(^ ^4^ ^)^.

A variety of mechanisms have been proposed to link red and processed meat consumption and the risk of CRC^(^ ^5^ ^)^. For red meat, in particular, it has been suggested that its high content of haem Fe is a substantial contributor^(^ ^6^ ^)^. Red meat undergoes fermentation in the colon that might alter the microbiota composition and result in the production of potentially genotoxic products that could play a role in oncogenesis. These agents include N-nitroso compounds (NOC), a complex mixture of nitrite-derived products formed either in processed meat itself or endogenously in the human gut via bacterial metabolism. NOC are alkylating agents that generate DNA adducts in human colonocytes after high red meat consumption^(^ ^7^ ^)^. We have recently shown that the level of the pro-mutagenic adduct O^6^-methyl-2-deoxyguanosine (O^6^MeG) is increased in murine colonocytes after consuming a diet high in red meat^(^ ^8^ ^)^. O^6^MeG is a known toxic and mutagenic base modification that, if unrepaired, can induce GC → AT transition mutations (typically found in the *K-ras* gene in human CRC)^(^ ^9^ ^)^ and also recombination events or mutations in the form of sister chromatid exchanges^(^ ^10^ ^)^. More recently, it has been suggested that high red meat consumption can increase the expression of certain oncogenic microRNA^(^ ^11^ ^)^.

Dietary fibre is a heterogeneous group of compounds, principally indigestible carbohydrates of plant origin that include NSP, starches that escape digestion in the small intestine (resistant starches, RS) and oligosaccharides. One possible mechanism for the reduction in the risk of CRC by dietary fibre is the production of SCFA via fermentation by the large-bowel microbiota^(^ ^12^ ^)^. Of the major SCFA, butyrate is of particular interest as it appears to be the preferred metabolic substrate for colonocytes, and butyrate also promotes a normal cellular phenotype. *In vitro* studies with CRC cell lines have shown that butyrate induces apoptosis^(^ ^13^ ^)^, reduces cell proliferation and promotes differentiation^(^ ^14^ ^)^. Animal experiments have shown that butyrate may reduce colorectal carcinogenesis by enhancing the apoptotic response to methylating carcinogens^(^ ^15^ ^,^ ^16^ ^)^.

Increasing large-bowel butyrate supply has the potential to improve colonic function and lower disease risk. RS is thought to be particularly effective in this regard as its fermentation generally favours butyrate production. Red meat and fibre (including RS) are generally consumed together as components of foods. Our animal studies have shown that dietary RS is able to oppose colonocyte DNA strand breaks, telomere shortening and pro-mutagenic DNA adduct formation in rodents fed red meat^(^ ^8^ ^,^ ^17^ ^,^ ^18^ ^)^. This protective effect correlated most closely with large-bowel butyrate levels, supporting a role for fermentation in risk modification. Acylated starches (classified as RS4, chemically modified), in which the acyl group is linked to the starch framework by an ester bond, can deliver specific SCFA to the large bowel where bacterial esterases release the SCFA. Ingestion of butyrylated high-amylose maize starch increases colonic butyrate levels in animals^(^ ^19^ ^)^ and humans^(^ ^20^ ^)^. Accordingly, the present study was carried out in healthy individuals to determine whether high red meat consumption generated O^6^MeG adducts in rectal epithelial cells, and whether concurrent consumption of high red meat and butyrylated high-amylose maize starch opposed this effect (primary aim). We also investigated the effects of these diets on other indices of colonic health including rectal proliferation, colonic fermentation products and microbiota composition, as these might participate in the generation of adducts.

## Methods

### Study design and participants

**Fig. 1:**
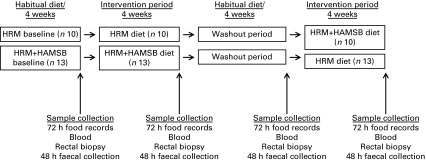
Overview of the randomised cross-over intervention study design. HRM, high red meat; HAMSB, butyrylated high-amylose maize starch.

The present study was conducted as a double-blind, randomised cross-over trial consisting of two intervention periods of 4 weeks each, preceded by a 4-week run-in (baseline) and separated by a 4-week (washout) period (Fig. 1). A group of twenty-three healthy volunteers participated in the trial. Exclusion criteria included evidence of active mucosal bowel disease, intolerance to high-fibre foods or any perceived contraindication to consumption of the test products. At enrolment, all participants showed no active bowel disease. During the entry (baseline) period, participants consumed their habitual diets. For the interventions, they were allocated randomly to a high red meat (HRM) diet or to a HRM diet supplemented with 40 g/d of butyrylated high-amylose maize starch (HRM+HAMSB diet). During the HRM intervention, participants consumed 300 g/d (raw weight) of cooked lean red meat that was supplied frozen in 100 g packs of lean mince, beef strips or lamb strips, with three packs to be consumed each day. During the HRM+HAMSB intervention, participants were also required to consume a total of two pre-packed 20 g sachets of HAMSB daily, one in the morning and one in the evening by mixing the powder into 250 ml of reduced-fat milk or orange juice. HAMSB was manufactured by Ingredion, whereby 23 % of each glucopyranosyl unit in high-amylose maize starch contained a butyrate molecule (degree of substitution 0·23) and was of the same batch as the product used previously^(^ ^21^ ^)^. Participants on the HRM arm of the study were asked to consume 250 ml of reduced-fat milk or orange juice per d to match the HRM+HAMSB intervention. During the intervention periods, participants reduced their intake of their habitual diet to accommodate the extra 300 g red meat. Participants were instructed to maintain their usual diet during the study but to avoid consuming high levels of protein or fibre, or probiotic supplements, except those prescribed for the study. Participants were also asked to avoid consuming, or record the use of, any medication that could interfere with bowel function (including antibiotics). Participants were monitored by a trial nurse (weekly) and dietitian (at the end of each 4-week period) to ensure that diet and intervention guidelines were followed, and weight was kept stable. Details of medical history and medications, weight, bowel health and symptoms, and adverse events were collected by the trial nurse throughout the study. Composition of the participants' diets and compliance with the interventions was assessed using weighed food diaries that were completed by the participants at the end of each 4-week dietary period, 3 d before each clinic visit. Food diaries were entered into FoodWorks Professional 7 Nutrition Calculation software (Xyris Software) by a dietitian, to calculate energy and macronutrient intake based on Australian food composition tables and food manufacturers' data. The present study was approved by the Flinders Clinical Research Ethics Committee (reference no. 155/09; Flinders Medical Centre, Bedford Park, SA, Australia), and all volunteers gave written informed consent. The present trial was registered in the Australian New Zealand Clinical Trials Registry as ACTRN12609000306213 (http://www.anzctr.org.au).

### Sample collection

Stool and rectal pinch biopsy samples were obtained at the completion of the 4-week entry period (baseline) and at the end of each intervention period. A complete faecal collection was made by all participants for the last 48 h of each dietary period, and the samples were stored in portable freezers ( − 20°C). At each visit to the Flinders Medical Centre clinic, an experienced gastroenterologist collected four rectal mucosal biopsies using alligator forceps through a 25 cm rigid sigmoidoscope; this procedure was performed without bowel preparation or prior dietary restriction. Biopsies were formalin-fixed and dehydrated through gradient alcohol and xylene before being embedded in paraffin wax.

### Stool analyses

Faecal samples were thawed at 4°C, pooled, homogenised, and then subsampled for analysis. For the determination of SCFA, weighed portions were diluted at 1:3 (w/w) with deionised water containing 1·68 mmol heptanoic acid/l as an internal standard (Sigma Chemical Co.), and processed for SCFA analysis using GC as described previously^(^ ^8^ ^)^. Total SCFA concentration was calculated as the sum of acetic, propionic, butyric, isobutyric, caproic, isovaleric and valeric acid concentrations. Total branched-chain fatty acids concentration was calculated as the sum of isobutyric and isovaleric acid concentrations. Phenol and *p-*cresols were measured in the faeces by using vacuum microdistillation and HPLC^(^ ^22^ ^)^. Faecal NH_3_ concentration was measured by using the indophenol blue method^(^ ^23^ ^)^. Aqueous extracts of the faeces were prepared by diluting 1 g faeces with 4 ml of distilled water, homogenised and centrifuged (4500 rpm, 4°C)^(^ ^24^ ^)^, and total apparent NOC were measured by chemical denitrosation with HBr and chemiluminescence detection of the released nitric oxide using a thermal energy analyser (TEA)^(^ ^25^ ^,^ ^26^ ^)^. Concentrations were calculated by comparing the TEA response of a faecal water sample with the response of an *N*-nitrosodipropylamine standard (16·6 μg/ml), and values were expressed as total apparent NOC (ng/ml)^(^ ^27^ ^)^.

### Rectal biopsy analysis

**Fig. 2:**
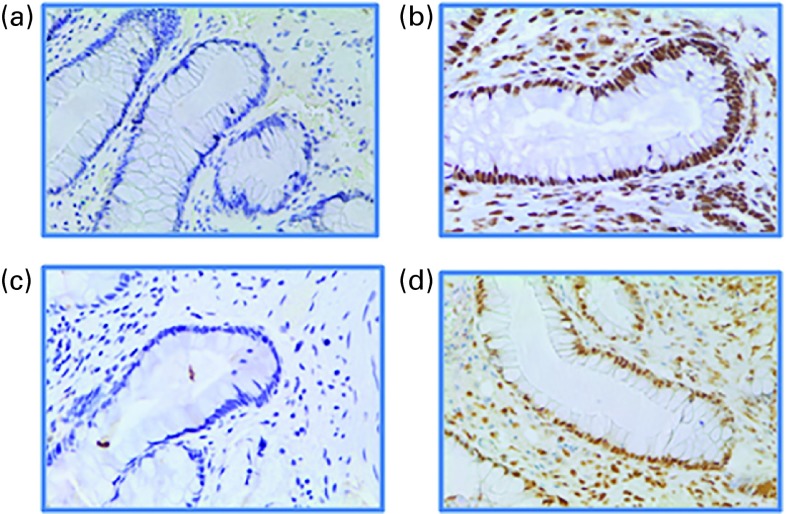
Light microscope images (20 ×  optical zoom) of human rectal crypts showing O^6^-methyl-2-deoxyguanosine staining intensity from the baseline and after the 4-week intervention phase selected from participant #20. Images showing the sections (a) at the end of the high red meat (HRM) baseline, (b) at the end of the 4-week HRM treatment, (c) at the end of the HRM+butyrylated high-amylose maize starch (HAMSB) baseline and (d) at the end of the 4-week HRM+HAMSB treatment.

The quantification of the O^6^MeG adduct load was performed using an immunohistochemical detection method^(^ ^8^ ^)^. The immunohistochemical measurement of O^6^MeG adducts has been previously used for many years mainly in different animal species^(^ ^8^ ^,^ ^28^ ^–^ ^32^ ^)^; however, this is the first time it has been applied to human colonic crypts. The specificity of the monoclonal antibody has been validated by RIA^(^ ^33^ ^)^. In brief, rectal biopsies were embedded in paraffin and sectioned at 4 μm, and their O^6^MeG adduct load was quantified using an anti-O^6^MeG antibody (Squarix Biotechnology); this antibody is listed as being specific for human tissue. Antigen retrieval (10 mm-citrate buffer) was performed, followed by RNase treatment (20 μl RNase A (10 mg/ml), Thermo Fisher Scientific; 5 μl RNase T (10 units/ml), Thermo Fisher; 100 μl PBS (pH 7·4) and stopped with a 5 min treatment with NaCl solution (140 mm)). DNA unwinding was achieved using alkali treatment (70 mm-NaOH/140 mm-NaCl, 1·5 ml) before applying Special Block A (Covance Laboratories) for 30 min. The O^6^MeG antibody (1:1000) was applied to the slides overnight at room temperature, followed by Special Block B (Covance Laboratories), before applying poly-horseradish peroxidase (HRP) anti-mouse IgG. Sections were counterstained with haematoxylin, and chromogen 3,3′-diaminobenzidine tetrahydrochloride (DAB) was used to visualise positive O^6^MeG staining. All slides were independently and randomly coded before quantification of nuclear staining for O^6^MeG with a computer image analysis protocol^(^ ^8^ ^)^. Overall, twenty appropriate crypts were visualised using an Olympus Micropublisher 3.3 RTV camera and Olysia Bio-report software (Olympus). Camera and microscope settings were calibrated before each image to ensure analytical consistency. To identify a linear path through a single row of nuclei along the crypt axis for all images taken, image analysis software developed by the CSIRO Mathematics Informatics and Statistics division, ‘Imview’ and ‘R for Windows’ 2.1.0, was used. Raw colour (red, green and blue), luminescence (*L*), normalised colour values (*r*= ¼ red/*L*, *g*= ¼ green/*L* and *b*= ¼ blue/*L*) and colour ratio (RoB = ¼ *r*/*b*) data points were calculated for each pixel along the length of the linear path. The number of cells within each half crypt was counted, and the calculated RoB ratio was then averaged for each nucleus within individual crypts. Total O^6^MeG values/crypt were achieved by summation of the ratio value for each nucleus along the crypt axis. Representative sections of one individual from each treatment group showing the immunohistochemical staining are shown in Fig. 2. Proliferation status of cells in the rectal crypts was determined by standard immunohistochemical techniques using the proliferating cell nuclear antigen (PCNA) antibody (PC10), as reported previously^(^ ^34^ ^,^ ^35^ ^)^. Slides were visualised by brown nuclear staining, and assessed as the number of Ki-67-positive cells/crypt.

### Molecular microbiology

Extraction of DNA from stool samples and subsequent quantitative real-time PCR (qPCR) was performed and analysed according to the method used by Christophersen and colleagues^(^ ^36^ ^)^. In brief, DNA was extracted using a repeat bead beating and column clean-up method, and qPCR assays amplified the 16S ribosomal RNA (rRNA) gene (or in the case of sulphate-reducing bacteria the adenosine-5′-phosphosulfate reductase (*aps*) gene) using primers that targeted bacterial species or groups of interest. The primer pairs and their amplification conditions are listed in online Supplementary Table S5. Data are expressed as absolute abundances and as a proportion of total bacteria. Bacterial targets were chosen for their relevance to gut health. In other words, we selected key species (e.g. *F. prausnitzii*) or groups of bacteria (e.g. *C.* *coccoides* group) that were responsible for the production of butyrate following fermentation of complex carbohydrates, particularly RS, by bacteria such *Ruminococcus bromii*. *P.* *distasonis* was chosen because of its potential role in the cleavage of butyrate from the butyrylated RS used in the study. We also examined changes in some potentially enteropathogenic species (e.g. *E.* *coli*), as well as in general groups such as *Lactobacillus* and *Bifidobacterium* that are regarded as markers of bowel health by many. A range of other bacteria such as those implicated in gut mucus barrier turnover and inflammatory bowel disease, such as *A. muciniphila*, were also targeted. Sulphate-reducing bacteria were included to determine whether the production of toxic hydrogen sulphide could contribute to large-bowel DNA adduct formation in response to red meat treatment.

### Statistical methods

All statistical analyses were performed with scripts in R, version 3.0.1, using the R statistical package^(^ ^37^ ^)^. Analyses were carried out using a linear mixed-effects model, with subjects as the random effect, on either base-10 logarithm-transformed data, where necessary, or on untransformed data for each of the variables.

Initial analyses were carried out using the two periods of the trial, including the baseline and washout periods. However, data analyses of the initial study showed that some of the response variables had carry-over effects, including the primary end-point O^6^MeG, epithelial proliferation, certain bacterial species but not SCFA (see the online Supplementary material for a full study dataset). This was indicated by a significant difference between the baseline level and the washout level of the response variable or a significant interaction between the week of diet consumption and the response variable. Therefore, the data analyses used in the present study were only those of the first period of the study (i.e. only measurement weeks 0 and 4). As a result, the analysis reported herein was carried out using only the baseline and the first-period data. The comparison between the groups in the first period of the trial was carried out using a linear mixed-effects model, testing for changes from the baseline and a difference between the treatments. For Tables 1–4, dietary intake, stool biochemistry, bacterial abundance (percentage of total bacteria) and rectal biology data for the first period of the trial are expressed as means with their standard errors of the mean for both groups (HRM and HRM+HAMSB), together with the increment and percentage change for each group. For each of these means, the significance of the change from the baseline is indicated. The final column of each table gives the *P* value for the difference between the two treatments at week 4, and these were tested using either the original data or the log 10-transformed data as appropriate.

**Table 1:** Dietary intake of the study participants during each diet period, based on 3 d weighed food records (Mean values with their standard errors; percentages)

	HRM group (*n* 10)	HRM+HAMSB group (*n* 13)†	
	Baseline	Week 4			Baseline	Week 4			
	Mean	sem	Mean	sem	Increment	Change (%)	Mean	sem	Mean	sem	Increment	Change (%)	*P* ‡
Energy (kJ/d)	9169	718	9463	613	+294	3	8578	421	9250	553	+672	7	0·98
Protein (g/d)	101	11	124*	5	+23	19	88	4	119**	7	+31	26	0·81
Fat (g/d)	80	10	77	9	–3	4	67	4	70	8	+3	4	0·80
Saturated fat (g/d)	31	5	34	4	+3	9	24	2	30	3	+6	20	0·75
Carbohydrate (g/d)	221	28	222	20	+1	1	244	17	256	16	+12	5	0·36
Sugar (g/d)	112	16	121	10	+9	7	120	12	129	9	+9	7	0·78
Starch (g/d)	108	16	99	17	–9	–9	122	9	125	9	+3	2	0·21
Fibre (g/d)	24	2	19**	2	–5	–26	28	3	29	3	+1	3	0·01§
Alcohol (g/d)	21	6	22	8	+1	5	11	2	7	2	–4	–57	0·52
Total Fe (mg/d)	13·6	0·8	15·1	0·8	+1·5	10	14·4	1·2	16·4	1·5	+2	12	0·69
Fe from meat (mg/d)	3·7	0·6	7·2***	0·9	+3·5	49	2·6	0·5	6·7***	0·4	4·1	61	0·56
Fe from non-meat (mg/d)	9·9	0·6	7·9	0·8	–2	–25	11·7	1·3	9·7	1·6	–2·0	21	0·92

HRM, high red meat; HAMSB, butyrylated high-amylose maize starch.

Mean value was significantly different from that at baseline: * *P*< 0·05, ** *P*< 0·01, *** *P*< 0·001 (linear mixed-effects model).

† HAMSB supplement contains 88 % total carbohydrate, approximately 20 % dietary fibre, 10 % moisture, < 1 % total fat and < 0·75 % protein.

‡ *P* value was obtained for treatment difference at week 4 (linear mixed-effects model).

§ *P*< 0·05.

**Table 2:** Effect of the dietary interventions in the first period on rectal biology (Mean values with their standard errors; percentages)

	HRM group (*n* 10)	HRM+HAMSB group (*n* 13)	
	Baseline	Week 4			Baseline	Week 4			
	Mean	sem	Mean	sem	Increment	Change (%)	Mean	sem	Mean	sem	Increment	Change (%)	*P* †
O^6^MeG load (staining intensity)	60·8	2·3	77·4**	5·8	16·6	21·4	59·8	3·2	67·5	2·3	7·7	11·4	0·14
PCNA (positive cells/crypt)	6·2	0·3	9·9***	1·0	+3·8	38	6·6	0·3	8·6*	0·7	+2·0	23	0·05‡

HRM, high red meat; HAMSB, butyrylated high-amylose maize starch; O^6^MeG, O^6^-methyl-2-deoxyguanosine; PCNA, proliferating cell nuclear antigen.

Mean value was significantly different from that at baseline: * *P*< 0·05, ** *P*< 0·01, *** *P*< 0·001 (linear mixed-effects model).

† *P* value was obtained for treatment difference at week 4 (linear mixed-effects model).

‡ *P*< 0·05.

**Table 3:** Effect of the dietary interventions in the first period on stool biochemistry (Mean values with their standard errors; percentages)

	HRM group (*n* 10)	HRM+HAMSB group (*n* 13)	
	Baseline	Week 4			Baseline	Week 4			
	Mean	sem	Mean	sem	Increment	Change (%)	Mean	sem	Mean	sem	Increment	Change (%)	*P* †
Faecal output (g/48 h)	266·4	42	264·7	46·7	–1·7	–0·6	226·8	50·1	293·6	36	66·8	22·8	0·61
Faecal pH	7·1	0·1	7·2	0·1	0·1	1·4	7·2	0·1	7·2	0·1	0	0	0·63
SCFA levels (mmol/48 h)													
Total	25·8	6·4	26·7	6·5	0·9	3·4	19·3	5·1	26·3*	4·1	7	26·6	0·48
Acetate	15·2	3·9	15·7	3·7	0·5	3·2	10·3	2·6	14·4*	2·3	4·1	28·5	0·61
Propionate	4·4	1·1	4·4	1·1	0	0	3·7	1·1	5·2*	0·9	1·5	28·8	0·27
Butyrate	4·3	1·1	4·3	1·1	0	0	3·6	1·2	4·7*	1	1·1	23·4	0·28
SCFA concentration (μmol/g)													
Total	86·5	10·1	91·6	10·2	5·1	5·6	73·2	8·3	89·3*	7·7	16·1	18	0·53
Acetate	50·8	6·2	54	5·4	3·2	5·9	39·7	4·2	48·9*	4·5	9·2	18·8	0·81
Propionate	14·4	2	14·9	2·2	0·5	3·4	13	1·6	16·9*	1·5	3·9	23·1	0·13
Butyrate	14·1	2	14·7	2·3	0·6	4·1	12·9	2·4	16·1	2·5	3·2	19·9	0·26
BCFA (μmol/g)	7·2	0·8	8·2	0·9	1	12·2	7·6	0·9	7·3	0·7	–0·3	–4·1	0·33
Phenol (μg/g)	1·2	0·3	0·7	0·1	–0·5	–71·4	1·3	0·4	1·6	1	0·3	18·8	0·63
*p*-Cresol (μg/g)	65·9	11·7	68·8	9·8	2·9	4·2	78·1	8·1	51·6**	10·1	–26·5	–51·4	0·02‡
NH_3_ (μmol/g)	20·4	2	16·1	1·5	–4·3	–26·7	17	1·6	15·5	1	–1·5	–9·7	0·93
NOC (ng/ml)	516	112	406·5	72	–109·5	–26·9	481·9	126·4	388·1	51·7	–93·8	–24·2	0·96

HRM, high red meat; HAMSB, butyrylated high-amylose maize starch; BCFA, branched-chain fatty acids; NOC, N-nitroso compounds.

Mean value was significantly different from that at baseline: * *P*< 0·05, ** *P*< 0·01, *** *P*< 0·001 (linear mixed-effects model).

† *P* value was obtained for treatment difference at week 4 (linear mixed-effects model).

‡ *P*< 0·05.

**Table 4:** Abundances of species and groups of bacteria (per g of stool and as a percentage of total bacteria)† (Mean values with their standard errors; percentages)

	HRM group (*n* 10)	HRM+HAMSB group (*n* 13)	
	Baseline	Week 4			Baseline	Week 4			
	Mean	sem	Mean	sem	Increment	Change (%)	Mean	sem	Mean	sem	Increment	Change (%)	*P* ‡
Per g of stool													
Total bacteria	3·8 × 10^9^	7·5 × 10^8^	4·7 × 10^9^	6·9 × 10^8^	+0·9 × 10^9^	19	4·0 × 10^9^	6·5 × 10^8^	5·4 × 10^9^	7·3 × 10^8^	+1·4 × 10^9^	26	0·56
* Clostridium coccoides* group	5·5 × 10^8^	1·2 × 10^8^	6·6 × 10^8^	8·5 × 10^7^	+1·1 × 10^8^	17	5·9 × 10^8^	0·9 × 10^8^	8·2 × 10^8^*	8·3 × 10^7^	+2·2 × 10^8^	28	0·33
* Clostridium* *leptum* group	5·3 × 10^8^	1·1 × 10^8^	7·5 × 10^8^	1·5 × 10^8^	+2·2 × 10^8^	29	5·1 × 10^8^	0·9 × 10^8^	9·2 × 10^8^*	1·6 × 10^8^	+4·1 × 10^8^	45	0·54
* Lactobacillu*s spp.	3·7 × 10^5^	1·3 × 10^5^	5·1 × 10^5^	1·0 × 10^5^	+1·4 × 10^5^	28	4·7 × 10^6^	3·6 × 10^6^	5·8 × 10^6^**	2·6 × 10^6^	+1·1 × 10^6^	19	0·26
* Parabacteroides distasonis*	1·4 × 10^7^	8·1 × 10^6^	1·2 × 10^7^	6·4 × 10^6^	–2·0 × 10^6^	17	9·0 × 10^6^	3·3 × 10^6^	2·4 × 10^8^***	7·9 × 10^7^	+2·3 × 10^8^	96	0·0004§§§
* Ruminococcus bromii*	9·0 × 10^6^	3·1 × 10^6^	9·7 × 10^6^	4·6 × 10^6^	+0·7 × 10^6^	7	1·8 × 10^7^	9·5 × 10^6^	3·6 × 10^7^*	1·0 × 10^7^	+1·8 × 10^7^	50	0·02§
* Ruminococcus torques*	2·2 × 10^7^	7·7 × 10^6^	2·1 × 10^7^	6·4 × 10^6^	–1·0 × 10^6^	5	2·2 × 10^7^	1·1 × 10^7^	0·52 × 10^7^*	0·25 × 10^7^	–1·7 × 10^7^	323	0·03§
Percentage of total bacteria													
* Escherichia coli*	1·08	0·82	1·5	1·04	+0·4	28	3·39	2·77	2·54**	2·35	–0·9	–34	0·02§
* P. distasonis*	0·27	0·16	0·2	0·11	–0·1	–35	0·34	0·18	4·37***	1·6	+4·0	92·2	0·0001§§§
* Ruminococcus gnavus*	0·3	0·12	0·31	0·14	0	0	0·45	0·14	0·22**	0·1	–0·2	–105	0·11
* R. torques*	1·11	0·55	0·72	0·36	–0·4	–54	0·98	0·58	0·15**	0·09	–0·8	–553	0·03§

HRM, high red meat; HAMSB, butyrylated high-amylose maize starch.

Mean value was significantly different from that at baseline: * *P*< 0·05, ** *P*< 0·01, *** *P*< 0·001 (linear mixed-effects model).

† As enumerated using quantitative real-time PCR and showing significant changes in response to the dietary treatments.

‡ *P* value was obtained for treatment difference at week 4 (linear mixed-effects model).

§ *P*< 0·05, §§ *P*< 0·01, §§§ *P*< 0·001.

The effects on the overall composition of the gut microbiota were analysed by combining all qPCR assays and performing a permutational-based multivariate analysis. Data were log-transformed before producing a resemblance matrix using Euclidean distance. Differences between the interventions were tested on first-period cross-sectional comparison only using Permanova^+^ version 1.06 (PRIMER-E). A *P* value < 0·05 was considered significant.

## Results

### Study comparisons for the whole dataset

Initial analyses were carried out using the two periods of the trial, including baseline and washout periods. However, data analyses of the initial study showed that some of the response variables had carry-over effects, including the primary end-point O^6^MeG, epithelial proliferation, certain bacterial species but not SCFA (see online Supplementary material for a full study dataset). There was a significant increase in the rectal crypt O^6^MeG adduct load when the participants consumed the HRM diet first compared with all the other intervention stages (*P*< 0·01; see online Supplementary Fig. S2(A)); however, when the participants consumed the HRM+HAMSB diet as the first intervention, there was no change in the O^6^MeG adduct load with the subsequent consumption of HRM (see online Supplementary Fig. S2(B)). There was a significant effect of treatment and treatment order on PCNA-positive cells/crypt (see online Supplementary Fig. S3(A) and (B)). For the participants on the HRM or HRM+HAMSB diet as their first intervention, the PCNA-positive cells significantly increased (*P*< 0·001). For those on the HRM or HRM+HAMSB diet (received as their first intervention), the positive cells significantly decreased after consuming their final treatment compared with the first treatment. Participants who consumed the HRM diet as the first treatment had significantly higher PCNA-positive cells/crypt compared with those who consumed the HRM diet as the second treatment (*P*< 0·001). We also observed that numbers of some bacteria in the washout phase were significantly different from those during the entry period or the dietary interventions (see online Supplementary Tables S3 and S4). The analysis revealed a diet-order effect on the microbiota composition. When the volunteers consumed the HRM+HAMSB diet as the first intervention, their microbiota composition significantly differed from that of the entry levels (*P*= 0·02), as well as from that of the subsequent HRM intervention (*P*= 0·02) and the washout levels (*P*= 0·005) in the same volunteers. Furthermore, the microbiota composition of these volunteers consuming the HRM+HAMSB diet first was also significantly different from that of those consuming HRM first (*P*= 0·01). However, when the volunteers consumed the HRM diet first, the microbial composition during the subsequent consumption of the HRM+HAMSB diet was only different from that of the washout levels (*P*= 0·02).

### Study comparisons for the first period

The results arising from the respective baseline periods and the first arm of the dietary intervention (i.e. at week 4 of the intervention) are described in detail below, as the results of the second arm (cross-over) showed carry-over effects for O^6^MeG, epithelial proliferation and certain bacterial species. The study was still adequately powered based on the primary end-point ‘O^6^MeG’. Calculations using a two-tailed *t* test with a power of 80 % with means of 60·8 and 77·4 and a standard deviation of 15 showed that group sizes of five were adequate to detect a difference between the baseline and the HRM intervention.

### Demographic data, participant characteristics and dietary intake

Recruitment commenced in July 2009, with each participant followed up for the 4-month duration of the interventions. Data collection was completed by September 2010. A total of twenty-five participants were assigned randomly, with twelve allocated to the HRM dietary intervention first and thirteen allocated to the HRM+HAMSB dietary intervention first. However, two participants withdrew before the commencement of the intervention diets; one due to unrelated medical problems and the other due to intolerance of the first rectal biopsy. Approximately one-third of the participants on the trial diets reported increased flatulence. Of the volunteers, ten (seven males and three females; mean age 62·1 (sem 1·8) years and mean body weight 79·8 (sem 5·6) kg) completed the HRM intervention as the first diet period, while thirteen (ten males and three females; age 62·7 (sem 1·7) years and body weight 82·4 (sem 3·5) kg) completed the HRM+HAMSB intervention first.

Participants maintained consistent body weight, with mean weights of 77·1 (sem 6·4) and 82·8 (sem 3·3) kg after the HRM and HRM+HAMSB interventions, respectively.

There was no difference between the diets for reported intake of energy, total and saturated fat, total carbohydrates and sugar, starch, alcohol or total Fe intake (Table 1). Compared with their respective baseline levels, protein intake was significantly increased by the HRM (*P*< 0·05) and HRM+HAMSB (*P*< 0·01) interventions. Fibre intake was decreased in the HRM group at week 4 compared with its baseline level (*P*< 0·01). Fibre intake was significantly lower in the HRM group than in the HRM+HAMSB group after 4 weeks (*P*< 0·01). Fe intake from meat was significantly higher for the HRM and HRM+HAMSB interventions at week 4 compared with their respective baseline levels (*P*< 0·001).

### Rectal epithelial measures

The O^6^MeG adduct load was increased at week 4 in the HRM group compared with its baseline level (*P*< 0·01); however, the addition of HAMSB to the HRM diet prevented this increase (Table 2). Relative to their respective baseline levels, the number of PCNA-labelled cells in the rectal epithelium increased for both the HRM (*P*< 0·001) and HRM+HAMSB (*P*< 0·05) groups; however, the number was lower following the HRM+HAMSB intervention than the HRM intervention (*P*< 0·05; Table 2).

### Stool biochemistry

The results of the stool analyses are presented in Table 3. Stool output and pH did not differ significantly between the treatments. Stool excretion of acetate, propionate, butyrate and total SCFA was higher in the HRM+HAMSB group at 4 weeks compared with its baseline level (*P*< 0·05) as was the stool concentrations of acetate, propionate and total SCFA (*P*< 0·05). Faecal *p-*cresol concentration was lower in the HRM+HAMSB group at 4 weeks compared with its baseline level (*P*< 0·01) and the HRM group (*P*< 0·05). Branched-chain fatty acids, phenols, NH_3_ and NOC were unaffected by the treatment.

### Stool bacteria

Significant changes in stool bacteria in response to the diet are presented in Table 4. When the numbers of bacteria/g of stool were examined, the HRM+HAMSB group at 4 weeks elicited an increase in the number of *Parabacteroides distasonis* relative to its baseline level (*P*< 0·0001) and the HRM group (*P*< 0·001). Compared with its baseline level, HRM+HAMSB consumption increased the numbers of *Lactobacillus* spp. (*P*< 0·01), the *Clostridium coccoides* group (*P*< 0·05), the *Clostridium leptum* group (*P*< 0·05) and *Ruminococcus bromii* (*P*< 0·05), but lowered the numbers of *Ruminococcus torques* (*P*< 0·05). When bacterial numbers were expressed as a percentage of total bacteria, the proportion of *P. distasonis* was increased by the consumption of the HRM+HAMSB diet at 4 weeks compared with its baseline level and the HRM group at 4 weeks (both *P*< 0·0001). Lower proportions of *Ruminococcus gnavus* (*P*< 0·01), *R.* torques (*P*< 0·01) and *E. coli* (*P*< 0·01) were evident in the HRM+HAMSB group at 4 weeks compared with its baseline level. The HRM+HAMSB group also had lower proportions of *R. torques* (*P*< 0·05) and *E. coli* (*P*< 0·05) than the HRM group at 4 weeks. When the results of all qPCR assays were combined and then analysed statistically to gain an indication of the impacts of the treatments on microbial diversity, it was found that the microbial diversity during the HRM+HAMSB intervention was different from that at baseline (*P*< 0·05) and during the HRM intervention (*P*< 0·01); however, the composition at baseline and the HRM intervention did not differ.

## Discussion

Previously, we reported that feeding a diet rich in red meat to rodents can increase the level of the pro-mutagenic DNA adduct (O^6^MeG) in the colon, whereas co-consumption of a fermentable carbohydrate can reduce this effect^(^ ^8^ ^)^. We have now shown that when free-living healthy human subjects consumed their normal habitual diet containing at least an additional 300 g red meat over a 4-week period, there was increased formation of the O^6^MeG adduct in the rectal epithelium. This increase in adduct formation might account, in part, for the increased risk of CRC associated with consuming high levels of red meat.

Studies in rodents have shown a positive correlation between cumulative O^6^MeG levels and tumour load^(^ ^38^ ^)^. This association is also supported, in humans, by the prevalence of higher O^6^MeG levels in DNA isolated from the distal region of the colon, where most sporadic CRC occurs^(^ ^39^ ^)^. The present study is the first to report on the effect of feeding a HRM diet to human subjects on the most predominant alkyl-induced DNA adduct O^6^MeG in the rectal epithelial tissue. In a randomised cross-over study comparing HRM, vegetarian and HRM/high-fibre diets, an increase in O^6^-carboxymethylguanine adduct levels was observed in exfoliated colonic epithelial cells isolated from the faeces of healthy volunteers consuming a HRM diet^(^ ^7^ ^)^. However, the relevance of DNA adducts in exfoliated cells to the *in situ* epithelial adduct load is unclear. Our findings show that such adducts do form in cells residing within the crypt and, thus, have the potential to form mutated clones that might progress to cancer.

The present study also confirms that dietary fermentable carbohydrate in the form of HAMSB can protect against red meat-induced colorectal DNA lesions in humans, and is consistent with epidemiological evidence that dietary fibre consumption reduces the risk of CRC. The present study and our previous work in rodents^(^ ^8^ ^,^ ^18^ ^,^ ^21^ ^,^ ^40^ ^)^ all point towards SCFA, particularly butyrate, to be the key mediators in preventing meat-induced DNA adducts and DNA strand breaks in colonic mucosa. Butyrate is a preferred metabolic substrate for colonocytes, and this SCFA has strong anti-tumorigenic properties *in vivo* and *in vitro* ^(^ ^15^ ^,^ ^41^ ^)^. In the present study, ingestion of HAMSB in conjunction with HRM was also able to favourably influence the colonic luminal environment, as evidenced by increased levels of SCFA and a reduction in the potentially toxic protein fermentation product *p-*cresol. This elevation of faecal butyrate with HAMSB confirms previous studies in human subjects^(^ ^20^ ^,^ ^42^ ^)^, and has the potential to improve colonic health and offer protection against CRC. Although consumption of a blend of types 2 and 3 RS in a recent human trial of hereditary CRC failed to reduce tumour incidence^(^ ^43^ ^)^, the relatively low daily intake of RS used in that study may have been insufficient to increase SCFA levels in stool (which were not measured). At least 20 g of RS/d may be needed to increase stool levels of SCFA^(^ ^44^ ^,^ ^45^ ^)^.

A reasonable explanation for the increase in O^6^MeG adducts with the HRM intervention is dietary haem. Haem is abundant in red meat, the majority of which derived from the diet passes into the large bowel^(^ ^46^ ^)^. We recently identified dietary haem as an agent that can increase O^6^MeG adducts in the colon of mice^(^ ^35^ ^)^. Haem Fe-rich meat has also been shown to increase alkylated DNA adducts in an *in vitro* digestion system^(^ ^47^ ^)^. Dietary haem may also increase the production of reactive oxygen species, causing cellular toxicity and pro-mutagenic lesions^(^ ^48^ ^,^ ^49^ ^)^. Other factors, such as bile acids^(^ ^50^ ^)^, could also contribute to adduct formation and DNA damage more broadly. Haem may also be responsible for the increased rectal cell proliferation in response to HRM and HRM+HAMSB consumption, as evidenced by more PCNA-labelled cells/crypt. Haem is associated with increased epithelial proliferation in the colon of rodents, and can injure the colonic surface epithelium by generating cytotoxic and oxidative stress^(^ ^6^ ^,^ ^51^ ^)^.

In the present study, we anticipated that NOC would increase in the stool of the participants consuming the HRM diet, and that this would explain a higher O^6^MeG adduct load. High dietary haem and red meat have previously been associated with increased luminal NOC in humans^(^ ^24^ ^,^ ^27^ ^,^ ^52^ ^)^. A dose–response relationship has been described between red meat intake and faecal NOC: low faecal NOC (374 μg/kg) at low red meat intake (60 g/d) and a 4- or 5-fold increase in faecal NOC with increased red meat intake of 240 and 420 g/d, respectively^(^ ^24^ ^)^. Lewin *et al.* ^(^ ^7^ ^)^ also observed an increase in faecal NOC in volunteers fed 420 g/d of red meat in comparison with a vegetarian diet, and suggested that NOC are important genotoxins involved in the generation of alkyl adducts^(^ ^7^ ^)^. Although we observed an increase in the O^6^MeG adduct load with HRM intake (300 g/d), we are unable to completely explain the lack of the effect of HRM on faecal NOC. One possible explanation is that the other studies^(^ ^7^ ^,^ ^24^ ^)^ had a very high level of control with the meals being consumed in an experimental facility. In the present study, we only controlled for the amount of meat in the diet so that other factors in the diet may account for the discrepancy.

There is growing recognition of both the importance of the large-bowel microbiota in human health and the strong role of diet in modulating its composition and metabolic activities. Using a suite of qPCR assays that targeted a range of bacteria important to gut health, we demonstrated significant shifts in the composition of the gut microbiota in response to the HRM+HAMSB, but not HRM, intervention. Our observation of an increase in the stool numbers of the *C. leptum* group and *R. bromii*, a member of the *C. leptum* group, in response to the consumption of RS as HAMSB is consistent with the effects previously observed in human subjects and with the central role that this bacterium appears to have in RS degradation^(^ ^53^ ^–^ ^55^ ^)^. This increase provides further evidence (additional to the observed increase in stool SCFA levels) that HAMSB was being consumed by the participants and was reaching the large bowel where it was available for fermentation. The numbers of *P. distasonis* were also increased by the HAMSB treatment, which is also consistent with changes in humans following consumption of butyrylated RS^(^ ^20^ ^)^. HAMSB is a chemically modified RS, and the forms of RS (classified as RS4) have been shown to be more likely to stimulate the growth of *P. distasonis* ^(^ ^53^ ^)^. In line with the HAMSB treatment, stool excretion of butyrate increased, primarily from the release of the bound butyrate; however, the higher number of bacteria in the *C. coccoides* group could also have contributed as a number of butyrate producers are classified in that group.

When the numbers of bacteria were expressed as a proportion of total bacteria, the addition of HAMSB to the diet also lowered the numbers of *E. coli* (a species with enteropathogenic variants and potential), *R. gnavus* and *R. torques* (numbers of which are high in the mucosa of some individuals with inflammatory bowel disease)^(^ ^56^ ^)^, supporting the potential of HAMSB to promote gut health. We did not observe any clear indication of the effects of HRM on the composition of the gut microbiota. However, the range of bacteria that we targeted is limited, and a more detailed analysis of populations may reveal changes. This may give an insight into the mechanisms of HRM-induced adduct formation and reasons for the associated increased risk of CRC. Furthermore, *in vitro* experiments have demonstrated that the formation of alkylated DNA adducts appear to depend on the microbial composition^(^ ^47^ ^)^. The changes that we have observed support the idea that the increases in stool SCFA levels and the associated protection against dietary HRM-mediated colorectal tissue damage that have occurred in response to dietary RS treatment are at least partly mediated by the gut microbiota, through both cleavage of the esterified butyrate and fermentation of the RS substrate. Part of the protective effect may also be attributable to reducing numbers and activities of bacteria with potential for harm.

A limitation of the present study was that the randomised cross-over design resulted in a period effect for the primary end-point ‘O^6^MeG adducts’. Participants allocated the HRM+HAMSB intervention in the first period did not have a HRM-induced increase in O^6^MeG adducts during the subsequent period. This contrasted with the increase in adduct formation that occurred when HRM was consumed first. This suggests that the consumption of HAMSB is able to protect against the damage caused by the HRM diet. We have presented in detail the analyses of the data from the first period (results incorporating both treatment periods are supplied in the online Supplementary material). Presentation of the data from the first arm reduces the statistical power of the study compared with that of the full cross-over. However, the primary end-point ‘O^6^MeG’ was still adequately powered, and the effect of HAMSB on SCFA (especially butyrate) is magnified. A further limitation of the study was that the right-sided colonic mucosa could not be evaluated, as biopsies were only taken from the rectum. This limits the application of the results to proximal colorectal carcinogenesis, particularly as genotypic differences between proximal and distal cancers exist^(^ ^57^ ^)^. More invasive studies are warranted to investigate whether the same effects observed in the rectum occur in other regions of the large bowel.

In summary, our findings show that high dietary red meat intake has detrimental effects on the colorectum by increasing pro-mutagenic DNA adducts and epithelial cell proliferation. Conversely, increasing luminal butyrate levels with HAMSB prevented the accumulation of O^6^MeG adducts. These findings might explain the increased risk for CRC associated with HRM consumption, and could point to a beneficial effect of butyrate-generating RS.

## Supplementary material

For supplementary material accompanying this paper visit http://dx.doi.org/10.1017/S0007114515001750. click here to view supplementary material

## References

[1] Jemal A, Center MM, DeSantis C, et al. (2010) Global patterns of cancer incidence and mortality rates and trends. Cancer Epidemiol Biomarkers Prev 19, 1893–1907. 20647400 10.1158/1055-9965.EPI-10-0437

[2] World Cancer Research Fund/American Institute for Cancer Research (2007) Food, Nutrition, Physical Activity, and the Prevention of Colon Cancer: A Global Perspective. Washington, DC: American Institute of Cancer Research.

[3] World Cancer Research Fund/American Institute for Cancer Research (2011) Continuous Update Project Report. Food, Nutrition, Physical Activity, and the Prevention of Colorectal Cancer. Washington, DC: American Institute of Cancer Research.

[4] Murphy N, Norat T, Ferrari P, et al. (2012) Dietary fibre intake and risks of cancers of the colon and rectum in the European prospective investigation into cancer and nutrition (EPIC). PLOS ONE 7, e39361. 22761771 10.1371/journal.pone.0039361 PMC3382210

[5] Bastide NM, Pierre FH & Corpet DE (2011) Heme iron from meat and risk of colorectal cancer: a meta-analysis and a review of the mechanisms involved. Cancer Prev Res (Phila) 4, 177–184. 21209396 10.1158/1940-6207.CAPR-10-0113

[6] Rijnierse A N IJ, de Wit N, et al. (2012) Dietary haem stimulates epithelial cell turnover by downregulating feedback inhibitors of proliferation in murine colon. Gut 61, 1041–1049. 21948946 10.1136/gutjnl-2011-300239

[7] Lewin MH, Bailey N, Bandaletova T, et al. (2006) Red meat enhances the colonic formation of the DNA adduct O6-carboxymethyl guanine: implications for colorectal cancer risk. Cancer Res 66, 1859–1865. 16452248 10.1158/0008-5472.CAN-05-2237

[8] Winter J, Nyskohus L, Young GP, et al. (2011) Inhibition by resistant starch of red meat-induced promutagenic adducts in mouse colon. Cancer Prev Res (Phila) 4, 1920–1928. 21885815 10.1158/1940-6207.CAPR-11-0176

[9] Lees NP, Harrison KL, Hall CN, et al. (2004) Reduced MGMT activity in human colorectal adenomas is associated with K-ras GC → AT transition mutations in a population exposed to methylating agents. Carcinogenesis 25, 1243–1247. 14963016 10.1093/carcin/bgh111

[10] Margison GP, Santibanez Koref MF & Povey AC (2002) Mechanisms of carcinogenicity/chemotherapy by O6-methylguanine. Mutagenesis 17, 483–487. 12435845 10.1093/mutage/17.6.483

[11] Humphreys KJ, Conlon MA, Young GP, et al. (2014) Dietary manipulation of oncogenic microRNA expression in human rectal mucosa: a randomized trial. Cancer Prev Res (Phila) 7, 786–795. 25092886 10.1158/1940-6207.CAPR-14-0053

[12] Topping DL & Clifton PM (2001) Short-chain fatty acids and human colonic function: roles of resistant starch and nonstarch polysaccharides. Physiol Rev 81, 1031–1064. 11427691 10.1152/physrev.2001.81.3.1031

[13] Medina V, Edmonds B, Young GP, et al. (1997) Induction of caspase-3 protease activity and apoptosis by butyrate and trichostatin A (inhibitors of histone deacetylase): dependence on protein synthesis and synergy with a mitochondrial/cytochrome c-dependent pathway. Cancer Res 57, 3697–3707. 9288776

[14] Bartram HP, Scheppach W, Schmid H, et al. (1993) Proliferation of human colonic mucosa as an intermediate biomarker of carcinogenesis: effects of butyrate, deoxycholate, calcium, ammonia, and pH. Cancer Res 53, 3283–3288. 8324739

[15] Clarke JM, Young GP, Topping DL, et al. (2012) Butyrate delivered by butyrylated starch increases distal colonic epithelial apoptosis in carcinogen-treated rats. Carcinogenesis 33, 197–202. 22080572 10.1093/carcin/bgr254 PMC3276328

[16] Le Leu RK, Brown IL, Hu Y, et al. (2003) Effect of resistant starch on genotoxin-induced apoptosis, colonic epithelium, and lumenal contents in rats. Carcinogenesis 24, 1347–1352. 12807738 10.1093/carcin/bgg098

[17] Toden S, Bird AR, Topping DL, et al. (2007) Dose-dependent reduction of dietary protein-induced colonocyte DNA damage by resistant starch in rats correlates more highly with caecal butyrate than with other short chain fatty acids. Cancer Biol Ther 6, 253–258. 17218781 10.4161/cbt.6.2.3627

[18] O'Callaghan NJ, Toden S, Bird AR, et al. (2012) Colonocyte telomere shortening is greater with dietary red meat than white meat and is attenuated by resistant starch. Clin Nutr 31, 60–64. 21963168 10.1016/j.clnu.2011.09.003

[19] Clarke JM, Topping DL, Bird AR, et al. (2008) Effects of high-amylose maize starch and butyrylated high-amylose maize starch on azoxymethane-induced intestinal cancer in rats. Carcinogenesis 29, 2190–2194. 18701436 10.1093/carcin/bgn192 PMC2577140

[20] Clarke JM, Topping DL, Christophersen CT, et al. (2011) Butyrate esterified to starch is released in the human gastrointestinal tract. Am J Clin Nutr 94, 1276–1283. 21940597 10.3945/ajcn.111.017228

[21] Conlon MA, Kerr CA, McSweeney CS, et al. (2012) Resistant starches protect against colonic DNA damage and alter microbiota and gene expression in rats fed a Western diet. J Nutr 142, 832–840. 22457395 10.3945/jn.111.147660 PMC3327741

[22] King RA, May BL, Davies DA, et al. (2009) Measurement of phenol and *p*-cresol in urine and feces using vacuum microdistillation and high-performance liquid chromatography. Anal Biochem 384, 27–33. 18848516 10.1016/j.ab.2008.09.034

[23] Chaney AL & Marbach EP (1962) Modified reagents for determination of urea and ammonia. Clin Chem 8, 130–132. 13878063

[24] Hughes R, Cross AJ, Pollock JR, et al. (2001) Dose-dependent effect of dietary meat on endogenous colonic N-nitrosation. Carcinogenesis 22, 199–202. 11159760 10.1093/carcin/22.1.199

[25] Bingham SA, Pignatelli B, Pollock JR, et al. (1996) Does increased endogenous formation of N-nitroso compounds in the human colon explain the association between red meat and colon cancer? Carcinogenesis 17, 515–523. 8631138 10.1093/carcin/17.3.515

[26] Russell WR, Gratz SW, Duncan SH, et al. (2011) High-protein, reduced-carbohydrate weight-loss diets promote metabolite profiles likely to be detrimental to colonic health. Am J Clin Nutr 93, 1062–1072. 21389180 10.3945/ajcn.110.002188

[27] Holtrop G, Johnstone AM, Fyfe C, et al. (2012) Diet composition is associated with endogenous formation of N-nitroso compounds in obese men. J Nutr 142, 1652–1658. 22833653 10.3945/jn.112.158824

[28] Zaidi NH, Potten CS, Margison GP, et al. (1993) Tissue and cell specific methylation, repair and synthesis of DNA in the upper gastrointestinal tract of Wistar rats treated with N-methyl-N'-nitro-N-nitrosoguanidine via the drinking water. Carcinogenesis 14, 1991–2001. 8222044 10.1093/carcin/14.10.1991

[29] Van Benthem J, Feron VJ, Leeman WR, et al. (1994) Immunocytochemical identification of DNA adducts, O6-methylguanine and 7-methylguanine, in respiratory and other tissues of rat, mouse and Syrian hamster exposed to 4-(methylnitrosamino)-1-(3-pyridyl)-1-butanone. Carcinogenesis 15, 2023–2029. 7522985 10.1093/carcin/15.9.2023

[30] Hong MY, Chapkin RS, Wild CP, et al. (1999) Relationship between DNA adduct levels, repair enzyme, and apoptosis as a function of DNA methylation by azoxymethane. Cell Growth Differ 10, 749–758. 10593651

[31] Hong MY, Lupton JR, Morris JS, et al. (2000) Dietary fish oil reduces O6-methylguanine DNA adduct levels in rat colon in part by increasing apoptosis during tumor initiation. Cancer Epidemiol Biomarkers Prev 9, 819–826. 10952099

[32] Nyskohus LS, Watson AJ, Margison GP, et al. (2013) Repair and removal of azoxymethane-induced O(6)-methylguanine in rat colon by O(6)-methylguanine DNA methyltransferase and apoptosis. Mutat Res 758, 80–86. 24140386 10.1016/j.mrgentox.2013.10.001

[33] Seiler F, Kamino K, Emura M, et al. (1997) Formation and persistence of the miscoding DNA alkylation product O6-ethylguanine in male germ cells of the hamster. Mutat Res 385, 205–211. 9506889 10.1016/s0921-8777(97)00043-8

[34] Le Leu RK, Hu Y, Brown IL, et al. (2010) Synbiotic intervention of *Bifidobacterium lactis* and resistant starch protects against colorectal cancer development in rats. Carcinogenesis 31, 246–251. 19696163 10.1093/carcin/bgp197

[35] Winter J, Young GP, Hu Y, et al. (2014) Accumulation of promutagenic DNA adducts in the mouse distal colon after consumption of heme does not induce colonic neoplasms in the western diet model of spontaneous colorectal cancer. Mol Nutr Food Res 58, 550–558. 24115497 10.1002/mnfr.201300430

[36] Christophersen CT, Morrison M & Conlon MA (2011) Overestimation of the abundance of sulfate-reducing bacteria in human feces by quantitative PCR targeting the *Desulfovibrio* 16S rRNA gene. Appl Environ Microbiol 77, 3544–3546. 21460115 10.1128/AEM.02851-10 PMC3126453

[37] Core Team (2013) R: A Language and Environment for Statistical Computing. Vienna, Austria: R Foundation for Statistical Computing http://www.R-project.org/.

[38] Povey AC (2000) DNA adducts: endogenous and induced. Toxicol Pathol 28, 405–414. 10862557 10.1177/019262330002800308

[39] Povey AC, Hall CN, Badawi AF, et al. (2000) Elevated levels of the pro-carcinogenic adduct, O(6)-methylguanine, in normal DNA from the cancer prone regions of the large bowel. Gut 47, 362–365. 10940272 10.1136/gut.47.3.362 PMC1728032

[40] Toden S, Bird AR, Topping DL, et al. (2006) Resistant starch prevents colonic DNA damage induced by high dietary cooked red meat or casein in rats. Cancer Biol Ther 5, 267–272. 16410726 10.4161/cbt.5.3.2382

[41] Le Leu RK, Hu Y & Young GP (2002) Effects of resistant starch and nonstarch polysaccharides on colonic luminal environment and genotoxin-induced apoptosis in the rat. Carcinogenesis 23, 713–719. 12016142 10.1093/carcin/23.5.713

[42] Clarke JM, Bird AR, Topping DL, et al. (2007) Excretion of starch and esterified short-chain fatty acids by ileostomy subjects after the ingestion of acylated starches. Am J Clin Nutr 86, 1146–1151. 17921395 10.1093/ajcn/86.4.1146

[43] Mathers JC, Movahedi M, Macrae F, et al. (2012) Long-term effect of resistant starch on cancer risk in carriers of hereditary colorectal cancer: an analysis from the CAPP2 randomised controlled trial. Lancet Oncol 13, 1242–1249. 23140761 10.1016/S1470-2045(12)70475-8

[44] Topping DL, Bird AR & Young GP (2009) Effect of aspirin or resistant starch on colorectal neoplasia in the Lynch syndrome. N Engl J Med 360, 1462, author reply 1462-1463. 19348022

[45] Worthley DL, Le Leu RK, Whitehall VL, et al. (2009) A human, double-blind, placebo-controlled, crossover trial of prebiotic, probiotic, and synbiotic supplementation: effects on luminal, inflammatory, epigenetic, and epithelial biomarkers of colorectal cancer. Am J Clin Nutr 90, 578–586. 19640954 10.3945/ajcn.2009.28106

[46] Young GP, Rose IS & St John DJ (1989) Haem in the gut. I. Fate of haemoproteins and the absorption of haem. J Gastroenterol Hepatol 4, 537–545. 2491221 10.1111/j.1440-1746.1989.tb00858.x

[47] Vanden Bussche J, Hemeryck LY, Van Hecke T, et al. (2014) O(6)-carboxymethylguanine DNA adduct formation and lipid peroxidation upon *in vitro* gastrointestinal digestion of haem-rich meat. Mol Nutr Food Res 58, 1883–1896. 24990219 10.1002/mnfr.201400078

[48] Glei M, Klenow S, Sauer J, et al. (2006) Hemoglobin and hemin induce DNA damage in human colon tumor cells HT29 clone 19A and in primary human colonocytes. Mutat Res 594, 162–171. 16226281 10.1016/j.mrfmmm.2005.08.006

[49] Knobel Y, Weise A, Glei M, et al. (2007) Ferric iron is genotoxic in non-transformed and preneoplastic human colon cells. Food Chem Toxicol 45, 804–811. 17157427 10.1016/j.fct.2006.10.028

[50] Ridlon JM, Kang DJ & Hylemon PB (2006) Bile salt biotransformations by human intestinal bacteria. J Lipid Res 47, 241–259. 16299351 10.1194/jlr.R500013-JLR200

[51] de Vogel J, Jonker-Termont DS, van Lieshout EM, et al. (2005) Green vegetables, red meat and colon cancer: chlorophyll prevents the cytotoxic and hyperproliferative effects of haem in rat colon. Carcinogenesis 26, 387–393. 15550456 10.1093/carcin/bgh331

[52] Lunn JC, Kuhnle G, Mai V, et al. (2007) The effect of haem in red and processed meat on the endogenous formation of N-nitroso compounds in the upper gastrointestinal tract. Carcinogenesis 28, 685–690. 17052997 10.1093/carcin/bgl192

[53] Martinez I, Kim J, Duffy PR, et al. (2010) Resistant starches types 2 and 4 have differential effects on the composition of the fecal microbiota in human subjects. PLoS ONE 5, e15046. 21151493 10.1371/journal.pone.0015046 PMC2993935

[54] Ze X, Duncan SH, Louis P, et al. (2012) *Ruminococcus bromii* is a keystone species for the degradation of resistant starch in the human colon. ISME J 6, 1535–1543. 22343308 10.1038/ismej.2012.4 PMC3400402

[55] Abell GC, Cooke CM, Bennett CN, et al. (2008) Phylotypes related to *Ruminococcus bromii* are abundant in the large bowel of humans and increase in response to a diet high in resistant starch. FEMS Microbiol Ecol 66, 505–515. 18616586 10.1111/j.1574-6941.2008.00527.x

[56] Png CW, Linden SK, Gilshenan KS, et al. (2010) Mucolytic bacteria with increased prevalence in IBD mucosa augment *in vitro* utilization of mucin by other bacteria. Am J Gastroenterol 105, 2420–2428. 20648002 10.1038/ajg.2010.281

[57] Iacopetta B (2002) Are there two sides to colorectal cancer? Int J Cancer 101, 403–408. 12216066 10.1002/ijc.10635

