# Supplementary material for: Butyrylated starch intake can prevent red meat-induced O6-methyl-2-deoxyguanosine adducts in human rectal tissue: a randomised clinical trial
Source: Br J Nutr. 2015 Jun 17;114(2):220–30. doi: 10.1017/S0007114515001750 (PMC4531472; doi:10.1017/S0007114515001750)
Supplement: Supplementary file 1 [file S0007114515001750sup001.docx]

**Supplemental Figure 1:** CONSORT diagram of participant flow for the high red meat and HAMSB randomised cross-over trial. HRM: high red meat intervention; HRM+HAMSB: high red meat and butyrylated high amylose maize starch intervention.


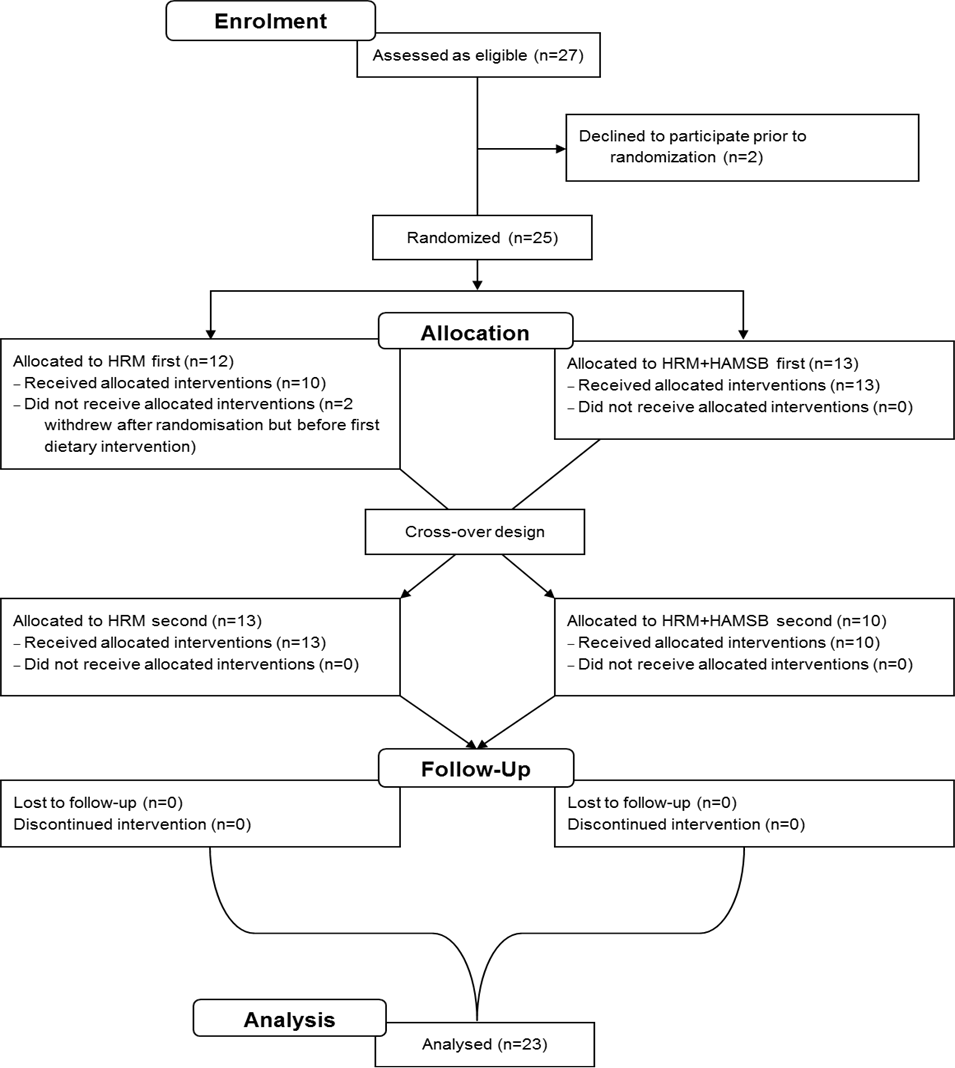


**Supplemental Figure 2.** Effect of diet and diet order on O^6^MeG adduct load in the rectal crypts. (A) Effect when individuals consumed the HRM treatment after entry. (B) Effect when the individuals consumed the HRM+HAMSB intervention after entry. Values with different superscript letters differ, P<0.05. *P*-values obtained from a Linear Mixed-Effects Model; HRM: high red meat; HRM+HAMSB: high red meat and butyrylated high amylose maize starch.


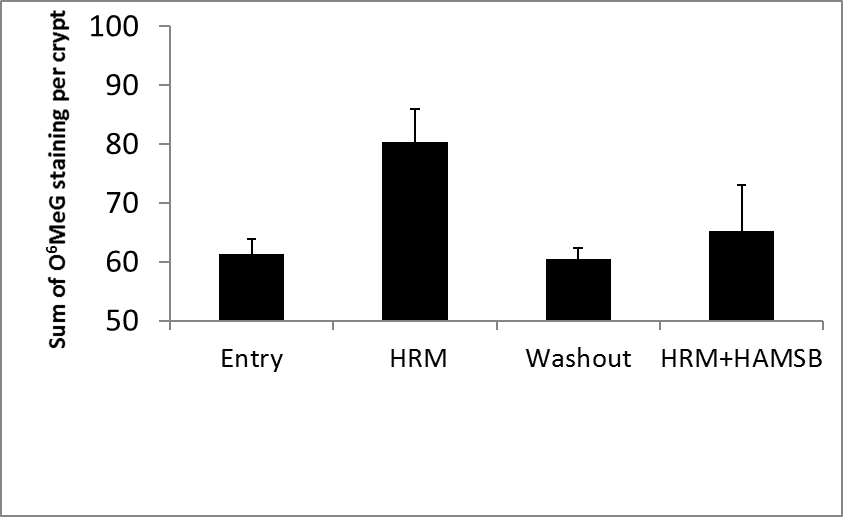


A

a

a

b

a


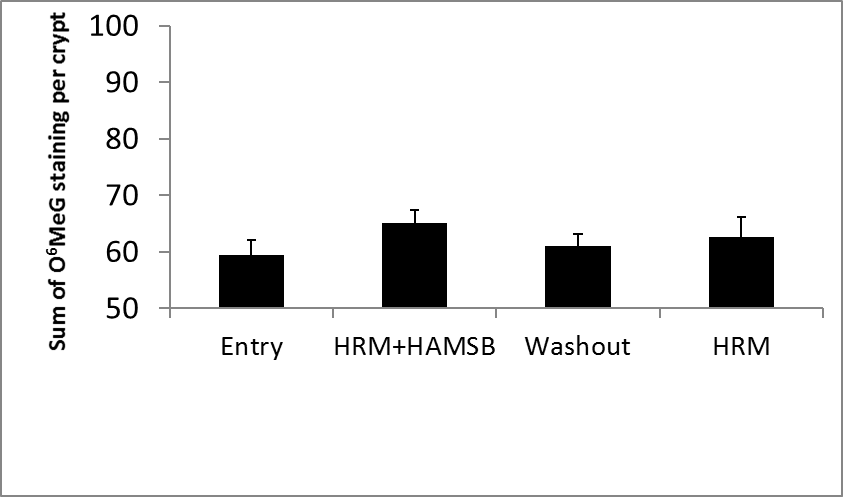


B

**Supplemental Figure 3.** Effect of diet and diet order on proliferation cell nuclear antigen (PCNA) labelled cells in the rectal crypts. (A) Effect when individuals consumed the HRM treatment after entry. (B) Effect when the individuals consumed the HRM+HAMSB treatment after entry. Bars (means ± SEM) in a row without a common superscript letter are significantly different (*P* < 0.05; Bonferroni adjusted *P*-values obtained from a Linear Mixed-Effects Model)


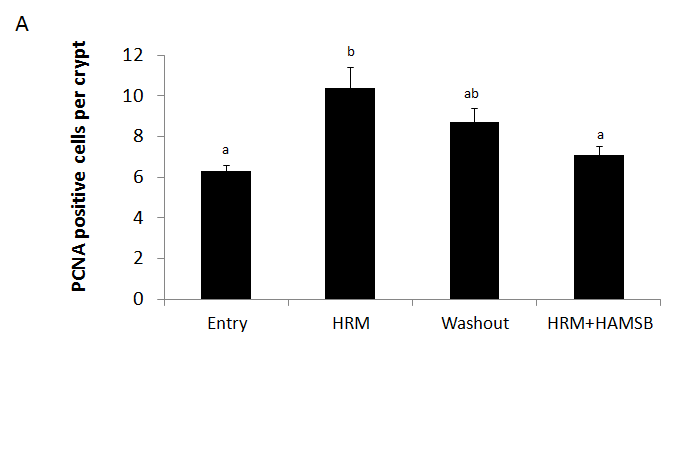

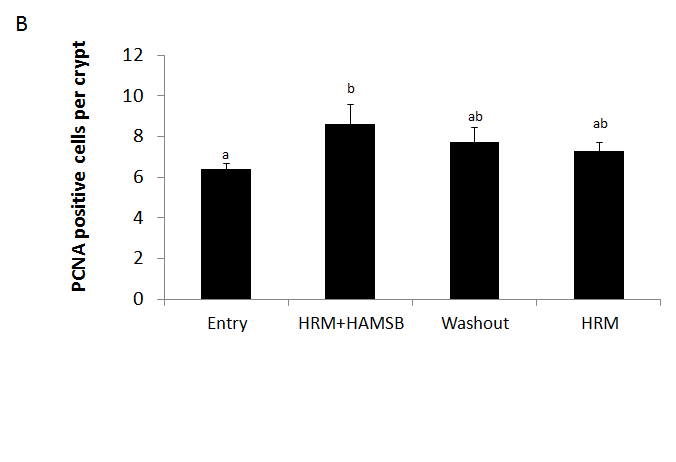


**Supplemental Figure 4.** Light microscope images (20 x optical zoom) of human rectal crypts showing O^6^MeG staining intensity from each treatment phase taken from one individual. (A) Representative section from Entry group, (B) representative section from HRM treatment, (C) representative section from washout phase, (D) representative section from HRM+HAMSB treatment; HRM: high red meat intervention; HRM+HAMSB: high red meat and butyrylated high amylose maize starch intervention.


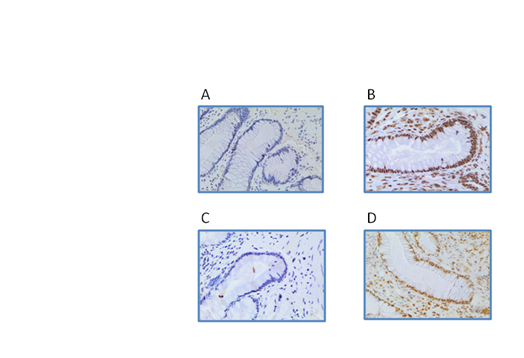


**Supplemental Table 1:** Dietary intake of study participants during each diet period, based on three-day weighed food records^1^

|  | **Entry** | **HRM** | **Washout** | **HRM+HAMSB** |
| --- | --- | --- | --- | --- |
| Energy (kJ/d) | 8835 ± 387 | 9167 ± 389 | 8781 ± 384 | 9116 ± 391 |
| Protein (g/d) | 93 ± 5^a^ | 124 ± 4^b^ | 90 ± 5^a^ | 119 ± 5^b^ |
| Fat (g/d) | 73 ± 5 | 77 ± 5 | 77 ± 5 | 68 ± 5 |
| Saturated fat (g/d) | 27 ± 3 | 32 ± 3 | 28 ± 2 | 30 ± 3 |
| Carbohydrate (g/d) | 234 ± 15 | 218 ± 12 | 222 ± 14 | 241 ± 12 |
| Sugar (g/d) | 117 ± 9 | 122 ± 8 | 106± 9 | 123 ± 8 |
| Starch (g/d) | 116 ± 8^b^ | 93 ± 10^a^ | 114 ± 10^b^ | 116 ± 7^b^ |
| Fibre (g/d) | 26 ± 2^b^ | 20 ± 2^a^ | 25 ± 2^b^ | 26 ± 2^b^ |
| Alcohol (g/d) | 15 ± 3 | 14 ± 4 | 16 ± 5 | 14 ± 3 |
| Total Iron (mg/d) | 14.1 ± 1.0 | 16.3 ± 1.0 | 12.5 ± 0.9 | 15.6 ± 1.2 |
| Iron from meat (mg/d) | 3.2 ± 0.8^a^ | 7.1± 0.9^b^ | 3.3 ± 0.6^a^ | 7.1 ± 0.5^b^ |
| Iron from non-meat (mg/d) | 10.9 ± 0.6 | 9.3 ± 0.8 | 9.3 ± 1.2 | 8.6 ± 1.6 |

^1^ Values (means ± SEM), rows in a row without a common superscript letter are significantly different (*P* < 0.05).

**Supplemental Table 2:** Effect of dietary intervention on stool biochemistry^1^

|  | **Entry** | **HRM** | **Washout** | **HRM + HAMSB** |
| --- | --- | --- | --- | --- |
| Faecal output (g/d) | 244 ± 33 | 241 ± 30 | 280 ± 32 | 309± 30 |
| Faecal pH | 7.1 ± 0.1 | 7.2 ± 0.1 | 7.2 ± 0.1 | 7.0 ± 0.1 |
| Acetate (µmol/g) | 45 ± 3.7^a^ | 51.0 ± 4.1^ab^ | 49± 4.2^ab^ | 57 ± 4.6^b^ |
| Propionate (µmol/g) | 14 ± 1.2^a^ | 15 ± 1.5^a^ | 15 ± 1.6^a^ | 20 ± 1.5^b^ |
| Butyrate (µmol/g) | 13 ± 1.6^a^ | 14 ± 1.9^a^ | 14 ±1.7^a^ | 21 ± 2.7^b^ |
| Total SCFA (µmol/g) | 80 ± 6.4^a^ | 88 ± 7.5^a^ | 86 ±7.2^a^ | 106 ± 8.3^b^ |
| BCFA | 4.1± 0.3^ab^ | 4.5 ± 0.4^b^ | 3.8 ± 0.3^a^ | 3.7 ± 0.3^a^ |
| Phenol (µg/g) | 1.3 ± 0.4^bc^ | 2.0 ± 1.2^ab^ | 1.4 ± 0.4^c^ | 1.3 ± 0.6^a^ |
| p-Cresol (µg/g) | 73 ± 7^b^ | 67 ± 5^b^ | 58 ± 6^ab^ | 50 ± 6^a^ |
| Ammonia (µmol/g) | 19 ± 1.3^b^ | 15 ± 1.0^a^ | 16 ± 1.3^ab^ | 16 ± 1.0^ab^ |
| NOC (µg/l NNO) | 497 ± 85 | 429± 51 | 472 ± 47 | 369± 36 |

^1^ Values (means ± SEM) in a row without a common superscript letter are significantly different (*P* < 0.05; Bonferroni adjusted *P*-values obtained from a Linear Mixed-Effects Model).

**Supplemental Table 3.** Abundances (per g of stool) of species and groups of bacteria as enumerated using QPCR.

|  | **Abundance (per g of stool)** | | | | | | | |
| --- | --- | --- | --- | --- | --- | --- | --- | --- |
|  | Entry | | HRM | | Washout | | HRM+HAMSB | |
|  | Mean | *SEM* | Mean | *SEM* | Mean | *SEM* | Mean | *SEM* |
| Total Bacteria | 3.88x10^9ab^ | 0.49x10^9^ | 4.08x10^9ab^ | 0.44x10^9^ | 3.32x10^9a^ | 0.49x10^9^ | 4.93x10^9b^ | 0.52x10^9^ |
| *A.muciniphila* | 5.14x10^7^ | 2.80x10^7^ | 5.41x10^7^ | 2.38x10^7^ | 2.55x10^7^ | 0.86x10^7^ | 3.48x10^7^ | 1.31x10^7^ |
| SRB_*aps* | 5.61x10^6^ | 1.62x10^6^ | 8.82x10^6^ | 3.15x10^6^ | 5.75x10^6^ | 2.80x10^6^ | 6.94x10^6^ | 2.15x10^6^ |
| *Bacteroides*-*Prevotella* | 3.87x10^8ab^ | 0.92x10^8^ | 4.67x10^8ab^ | 1.15x10^8^ | 3.30x10^8a^ | 1.19x10^8^ | 5.27x10^8b^ | 0.96x10^8^ |
| *P.distasonis* | 1.12x10^7b^ | 0.40x10^7^ | 1.41x10^7b^ | 0.63x10^7^ | 2.05x10^6a^ | 0.64x10^6^ | 1.74x10^8c^ | 0.53x10^8^ |
| *B.fragilis* group | 1.56x10^8ab^ | 0.33x10^8^ | 1.99x10^8ab^ | 0.54x10^8^ | 9.85x10^7a^ | 2.79x10^7^ | 2.20x10^8b^ | 0.44x10^8^ |
| *Bifidobacterium* spp. | 2.09x10^7ab^ | 0.42x10^7^ | 3.02x10^7ab^ | 1.04x10^7^ | 2.37x10^7a^ | 0.62x10^7^ | 3.75x10^7b^ | 1.09x10^7^ |
| *Clostridium* cluster I | 2.92x10^6^ | 0.76x10^6^ | 3.97x10^6^ | 0.98x10^6^ | 3.86x10^6^ | 1.46x10^6^ | 4.30x10^6^ | 1.11x10^6^ |
| *C.coccoides* group | 5.74x10^8ab^ | 0.71x10^8^ | 5.87x10^8ab^ | 0.71x10^8^ | 4.67x10^8a^ | 0.66x10^8^ | 7.24x10^8b^ | 0.66x10^8^ |
| *C.leptum* group | 5.18x10^8ab^ | 0.69x10^8^ | 6.56x10^8ab^ | 1.11x10^8^ | 4.78x10^8a^ | 0.88x10^8^ | 8.18x10^8b^ | 1.11x10^8^ |
| *E.coli* | 1.36x10^8^ | 1.14x10^8^ | 1.13x10^8^ | 0.78x10^8^ | 2.00x10^8^ | 1.62x10^8^ | 1.01x10^8^ | 0.91x10^8^ |
| *E.faecium* | 1.04x10^8^ | 1.06x10^8^ | 3.04x10^5^ | 1.40x10^5^ | 2.06x10^6^ | 1.96x10^6^ | 2.41x10^6^ | 2.03x10^6^ |
| *Enterococcus* spp. | 1.59x10^8c^ | 1.56x10^8^ | 2.02x10^6ab^ | 0.58x10^6^ | 1.18x10^6a^ | 0.43x10^6^ | 8.13x10^6bc^ | 2.92x10^6^ |
| *F.prausnitzii* | 3.46x10^8^ | 0.63x10^8^ | 4.25x10^8^ | 0.85x10^8^ | 3.32x10^8^ | 0.70x10^8^ | 4.55x10^8^ | 0.78x10^8^ |
| *Lactobacillu*s spp. | 2.81x10^6^ | 2.08x10^6^ | 1.87x10^6^ | 0.94x10^6^ | 1.06x10^6^ | 0.39x10^6^ | 3.43x10^6^ | 1.60x10^6^ |
| *Prevotella* | 6.91x10^7^ | 4.62x10^7^ | 8.24x10^7^ | 5.41x10^7^ | 1.36x10^8^ | 1.00x10^8^ | 6.86x10^7^ | 3.85x10^7^ |
| *R.bromii* | 1.41x10^7a^ | 0.56x10^7^ | 8.71x10^6a^ | 2.52x10^6^ | 7.53x10^6a^ | 2.38x10^6^ | 2.71x10^7b^ | 0.65x10^7^ |
| *R.gnavus* | 9.90x10^6^ | 2.26x10^6^ | 1.65x10^7^ | 0.52x10^7^ | 1.92x10^7^ | 0.84x10^7^ | 8.57x10^6^ | 2.17x10^6^ |
| *Roseburia* spp. | 4.35x10^8^ | 0.76x10^8^ | 4.40x10^8^ | 0.88x10^8^ | 4.18x10^8^ | 0.68x10^8^ | 4.30x10^8^ | 0.54x10^8^ |
| *R.torques* | 2.21x10^7^ | 0.73x10^7^ | 1.87x10^7^ | 0.47x10^7^ | 1.35x10^7^ | 0.39x10^7^ | 8.67x10^6^ | 2.35x10^6^ |

Data are presented as the mean and standard error of the mean (SEM). Means in a row without a common superscript letter are significantly different (*P* < 0.05; Bonferroni adjusted *P*-values obtained from a Linear Mixed-Effects Model).

**Supplemental Table 4.** Relative microbial abundances (as a percentage of total stool bacteria).

Data are presented as the mean and standard error of the mean (SEM). Means in a row without a common superscript letter are significantly different (*P* < 0.05; Bonferroni adjusted *P*-values obtained from a Linear Mixed-Effects Model).

**Supplementary Table 5**: Quantitative real-time PCR primers and their amplification conditions.

| Target | Primers | Sequence (5’-3’) | Conc. (nM) | Annealing | | Reference |
| --- | --- | --- | --- | --- | --- | --- |
|  |  |  |  | Temp (°C) | Time (Sec.) |  |
| *Akkermansia muciniphila* | AM1  AM2 | CAGCACGTGAAGGTGGGGAC  CCTTGCGGTTGGCTTCAGAT | 350 | 63 | 30 | ([1](#_ENREF_1)) |
| *Bifidobacterium* spp. | Bif-F  Bif-R | TCGCGTC(C/T)GGTGTGAAAG  CCACATCCAGC(A/G)TCCAC | 600 | 56 | 20 | ([2](#_ENREF_2)) |
| *Clostridium coccoides group* | g-Ccoc-F  g-Ccoc-R | AAATGACGGTACCTGACTAA  CTTTGAGTTTCATTCTTGCGAA | 500 | 60 | 20 | ([3](#_ENREF_3)) |
| *Clostridium leptum group* | sg-Clept-F  sg-Clept-R | CTTTGAGTTTCATTCTTGCGAA  GCACAAGCAGTGGAGT | 500 | 56 | 20 | ([3](#_ENREF_3)) |
| SRB^1^_*aps*^2^ | APS3F  APS2R | TGGCAGATCATGWTYAAYGG  GGGCCGTAACCRTCYTTRAA | 400 | 56 | 30 | ([4](#_ENREF_4)) |
| *Clostridium cluster I* | C.clu F  C.clu R | TACCHRAGGAGGAAGCCAC  GTTCTTCCTAATCTCTACGCAT | 500 | 63 | 20 | ([5](#_ENREF_5)) |
| *Enterocuccus spp.* | Entero F  Entero R | CCCTTATTGTTAGTTGCCATCATT  ACTCGTTGTACTTCCCATTGT | 600 | 59 | 15 | ([6](#_ENREF_6)) |
| *Escherichia coli* | E.coli F  E.coli R | CATGCCGCGTGTATGAAGAA  CGGGTAACGTCAATGAGCAAA | 300 | 60 | 20 | ([7](#_ENREF_7)) |
| *Ruminococcus bromii* | He-10F  He-10R | GGTCTTGACATCCAACTAACGAAGT  TTTTGTCAACGGCAGTCCTAT | 500 | 60 | 30 | ([8](#_ENREF_8)) |
| *Enterococcus faecium* | Efm07  Efm08 | AAGTCGAACGCTTCTTTTTCCA  CCAAGTGTTATCCCCTTCTGATG | 500 | 62 | 30 | ([9](#_ENREF_9)) |
| *Ruminococcus gnavus* | RgnaF  RgnaR | GGACTGCATTTGGAACTGTCAG  AACGTCAGTCATCGTCCAGAAAG | 500 | 58 | 20 | ([10](#_ENREF_10)) |
| *Ruminococcus torques* | RtorF  RtorR | GCTTAGATTCTTCGGATGAAGAGGA  AGTTTTTACCCCCGCACCA | 500 | 58 | 40 | ([10](#_ENREF_10)) |
| *Bacteroides-Prevotella* group | Bac-preF  Bac-preR | CTGAACCAGCCAAGTAGCG  CCGCAAACTTTCACAACTGACTTA | 700 | 58 | 45 | ([11](#_ENREF_11)) |
| *Bacteroides fragilis* group | Bfr-F  Bfr-R | CTGAACCAGCCAAGTAGCG  CCGCAAACTTTCACAACTGACTTA | 500 | 56 | 20 | ([12](#_ENREF_12)) |
| *Faecalibacterium prausnitzii* | FPR-1F  FPR-2R | AGATGGCCTCGCGTCCGA  CCGAAGACCTTCTTCCTCC | 500 | 60 | 20 | ([13](#_ENREF_13)) |
| *Lactobacillus group* | Lacto-F  Lacto-R | AGCAGTAGGGAATCTTCCA  CACCGCTACACATGGAG | 600 | 56 | 20 | ([14](#_ENREF_14), [15](#_ENREF_15)) |
| *Prevotella* spp. | PreF  PreR | CACCAAGGCGACGATCA  GGATAACGCCYGGACCT | 700 | 56 | 45 | ([16](#_ENREF_16)) |
| *Parabacteroides distasonis* | BdisF  BdisR | TGATCCCTTGTGCTGCT  ATCCCCCTCATTCGGA | 400 | 60 | 20 | ([12](#_ENREF_12)) |
| *Roseburia* spp. | RosF  RosR | TACTGCATTGGAAACTGTCG  CGGCACCGAAGAGCAAT | 700 | 58 | 45 | ([16](#_ENREF_16)) |
| *Total bacteria^*^* | 338F  518R | ACTCCTACGGGAGGCAGCAG  ATTACCGCGGCTGCTGG | 500 | 53 | 15 | ([17](#_ENREF_17)) |

^*^ 3 step PCR with a 72˚C step for 20 s.

^1^Sulfate-reducing bacteria, ^2^Adenosine-5-phosphosulfate reductase gene

**Supplemental References:**

1. Collado MC, Derrien M, Isolauri E, de Vos WM, Salminen S. Intestinal Integrity and Akkermansia muciniphila, a Mucin-Degrading Member of the Intestinal Microbiota Present in Infants, Adults, and the Elderly. Appl Environ Microbiol 2007;73(23):7767-70.

2. Rinttilä T, Kassinen A, Malinen E, Krogius L, Palva A. Development of an extensive set of 16S rDNA-targeted primers for quantification of pathogenic and indigenous bacteria in faecal samples by real-time PCR. Appl Microbiol 2004;97(6):1166-77.

3. Matsuki T, Watanabe K, Fujimoto J, Takada T, Tanaka R. Use of 16S rRNA Gene-Targeted Group-Specific Primers for Real-Time PCR Analysis of Predominant Bacteria in Human Feces. Appl Environ Microbiol 2004;70(12):7220-8.

4. Christophersen CT, Morrison M, Conlon MA. Abundance of sulphate-reducing bacteria in human feces is overestimated by QPCR that targets the Desulfovibrio 16S rRNA gene. Appl Environ Microbiol 2011;77(10):3544-6.

5. Song Y, Liu C, Finegold SM. Real-time PCR quantitation of clostridia in feces of autistic children. Appl Environ Microbiol 2004;70(11):6459-65.

6. Rinttila T, Kassinen A, Malinen E, Krogius L, Palva A. Development of an extensive set of 16S rDNA-targeted primers for quantification of pathogenic and indigenous bacteria in faecal samples by real-time PCR. J Appl Microbiol 2004;97(6):1166-77.

7. Huijsdens XW, Linskens RK, Mak MT, Meuwissen SGM, Vandenbroucke-Grauls C, Savelkoul PHM. Quantification of bacteria adherent to gastrointestinal mucosa by real-time PCR. J Clin Microbiol 2002;40(12):4423-7.

8. Mondot S, Kang S, Furet JP, et al. Highlighting New Phylogenetic Specificities of Crohn's Disease Microbiota. Inflammatory Bowel Diseases 2011;17(1):185-92.

9. Firmesse O, Rabot S, Bermúdez-Humarán LG, Corthier G, Furet JP. Consumption of Camembert cheese stimulates commensal enterococci in healthy human intestinal microbiota. FEMS Microbiology Letters 2007;276(2):189-92.

10. Png CW, Linden SK, Gilshenan KS, et al. Mucolytic Bacteria With Increased Prevalence in IBD Mucosa Augment In Vitro Utilization of Mucin by Other Bacteria. Am J Gastroenterol 2010;105(11):2420-8.

11. Bartosch S, Fite A, Macfarlane GT, McMurdo MET. Characterization of bacterial communities in feces from healthy elderly volunteers and hospitalized elderly patients by using real-time PCR and effects of antibiotic treatment on the fecal microbiota. Appl Environ Microbiol 2004;70(6):3575-81.

12. Liu C, Song Y, McTeague M, Vu AW, Wexler H, Finegold SM. Rapid identification of the species of the Bacteroides fragilis group by multiplex PCR assays using group- and species-specific primers. FEMS Microbiol Lett 2003;222(1):9-16.

13. Wang R-F, Cao W-W, Cerniglia CE. Phylogenetic Analysis of Fusobacterium prausnitzii Based upon the 16S rRNA Gene Sequence and PCR Confirmation. Int J Syst Bacteriol 1996;46(1):341-3.

14. Heilig HGHJ, Zoetendal EG, Vaughan EE, Marteau P, Akkermans ADL, de Vos WM. Molecular Diversity of Lactobacillus spp. and Other Lactic Acid Bacteria in the Human Intestine as Determined by Specific Amplification of 16S Ribosomal DNA. Appl Environ Microbiol 2002;68(1):114-23.

15. Walter J, Hertel C, Tannock GW, Lis CM, Munro K, Hammes WP. Detection of Lactobacillus, Pediococcus, Leuconostoc, and Weissella Species in Human Feces by Using Group-Specific PCR Primers and Denaturing Gradient Gel Electrophoresis. Appl Environ Microbiol 2001;67(6):2578-85.

16. Larsen N, Vogensen FK, van den Berg FWJ, et al. Gut Microbiota in Human Adults with Type 2 Diabetes Differs from Non-Diabetic Adults. PLoS ONE 2010;5(2):e9085.

17. Fierer N, Jackson JA, Vilgalys R, Jackson RB. Assessment of Soil Microbial Community Structure by Use of Taxon-Specific Quantitative PCR Assays. Appl Environ Microbiol 2005;71(7):4117-20.
